# Pituitary Stalk Germ Cell Tumors: Retrospective Case Series and Literature Review

**DOI:** 10.1155/2022/9213220

**Published:** 2022-03-26

**Authors:** Han Chen, Ming Ni, Yun Xu, Li-Yong Zhong

**Affiliations:** ^1^Department of Endocrinology, Beijing Tiantan Hospital, Capital Medical University, Beijing 100070, China; ^2^Department of Neurosurgery, Beijing Tiantan Hospital, Capital Medical University, Beijing 100070, China; ^3^Department of Obstetrics and Gynaecology, Beijing Tiantan Hospital, Capital Medical University, Beijing 100070, China

## Abstract

**Objective:**

Intracranial germ cell tumors with isolated pituitary stalk involvement are rare. Early recognition and long-term monitoring deserve further exploration.

**Methods:**

A retrospective study reviewing eleven intracranial germ cell tumor patients with isolated pituitary stalk involvement was performed.

**Results:**

Seven boys and four girls who presented with a hyperintense pituitary stalk on postcontrast T1-weighted magnetic resonance imaging without a posterior pituitary signal were included. The average maximum width of the pituitary stalk was 5.2 ± 1.6 mm. Polydipsia and polyuria occurred in all patients, followed by growth retardation, fatigue, and amenorrhoea. Eight patients (72%) had concomitant partial anterior pituitary hormone deficiency. Seven patients initially had elevated human chorionic gonadotropin levels. After chemoradiotherapy, ten patients attended follow-up. Central diabetes insipidus remained in all survivors, and four (40%) of them had concomitant partial anterior pituitary hormone deficiency, primarily of growth hormone and insulin-like growth factor-1. The causes of the delayed diagnosis of previous studies were mainly negative tumor markers and the initial pathological diagnosis of autoimmune diseases.

**Conclusion:**

Isolated pituitary stalk lesions could be a signal of intracranial germ cell tumors, especially coexisting with diabetes insipidus, hypopituitarism, and a worse response to glucocorticoid therapy. Negative results of tumor markers and pathology could not exclude the diagnosis. Chemoradiotherapy is an effective therapy, leaving mild-t-moderate hypothalamus-pituitary dysfunction. This rare neuroimaging feature may be used as a factor to predict long-term neuroendocrine outcomes.

## 1. Introduction

Intracranial germ cell tumors (iGCTs) are malignant neoplasms [1, 2] that are common in children and young adolescents [3]. IGCTs are considered a mismigrated progenitor during early embryogenesis, with distribution along the midline of body development [4] The hypothalamic-pituitary region is the second primary site of iGCTs after the pineal region, and a solitary suprasellar lesion is possible. Compared to an enlarged pituitary, suprasellar mass, hypothalamic involvement, and synchronous bifocal lesions, iGCTs with isolated pituitary stalk involvement are rare and difficult to diagnose, accounting for 13% of pediatric idiopathic pituitary stalk involvement [5–7] and/or diabetes insipidus cases [8]. However, identifying the underlying condition often requires a long period of observation, which can worsen nervous system damage and pituitary defects and increase the risk of dissemination [9–11].

Generally, the diagnostic flow charts and the treatment strategies for pituitary stalk involvement caused by iGCTs are described as follows. (1) Consideration of pathological thickening of the pituitary stalk: the imaging measurement of the pituitary stalk was 3–4 mm or more, indicating potential pathological thickening [8]. The coexistence of diabetes insipidus and hypopituitarism supports the diagnosis of neoplastic diseases [8, 12]. (2) Consideration of germ cell tumors: significant hyperintense intrasellar or pituitary stalk on postcontrast T1-weight magnetic resonance imaging (MRI) with an absent bright spot in the posterior pituitary lobe suggested the possible diagnosis of iGCT. Elevated concentrations of human chorionic gonadotropin (hCG) and alpha-fetoprotein (AFP) in serum and/or cerebrospinal fluid (CSF) are specific evidence. Pathological examination is the gold standard, and a good response to chemoradiotherapy is a supplementary diagnosis. (3) Treatment of germ cell tumors: as a local lesion, whole-brain or whole-ventricle radiation with adjuvant chemotherapy has been considered the optimized therapeutic strategy for iGCTs with isolated pituitary involvement. After tumors are removed, sequelae should also be highlighted. Theoretically, chemoradiotherapy without surgical damage to the pituitary gland and pituitary stalk may preserve the partial neuroendocrine function of patients with isolated pituitary stalk involvement. However, research has thus far proven inconclusive.

The clinical management of iGCTs with isolated pituitary stalk involvement remains questionable as follows: (1) How can germinomas be distinguished from other pituitary stalk lesions to decrease the risk of progression or dissemination? (2) Combined with rarely isolated pituitary stalk germ cell tumors, what is the role of the negative tumor marker? (3) Can the diagnosis of iGCTs be ruled out based on the pathological result of autoimmune diseases? (4) Would survivors of isolated pituitary stalk involvement need long-term or even lifelong multiple hormone replacements as common sellar/suprasellar disease? To better describe iGCTs with isolated pituitary stalk involvement, this retrospective study was performed. Previously published cases and updated management were also reviewed. Published studies related to the management of iGCTs with pituitary stalk lesions and advanced multidisciplinary therapy were also analyzed.

## 2. Materials and Methods

The present retrospective study was conducted in accordance with the Declaration of Helsinki for human research on humans, and the protocol was approved by the Ethics Committee of Beijing Tiantan Hospital affiliated with Capital Medical University (Beijing, China).

Medical records of eleven outpatients with iGCT with a radiographic presentation of isolated pituitary stalk involvement diagnosed at our institution between 2015 and 2021 were collected for every outpatient visit. The inclusion criteria were as follows: (1) isolated pituitary stalk involvement was defined as a width of the pituitary stalk of ＞3 mm or a mass on the pituitary stalk on the MRI scan; (2) postcontrast T1-weighted MRI was received; (3) germ cell tumors were confirmed by elevated tumor markers (hCG>2.6 mIU/ml and/or AFP>7 ng/ml), good response to chemotherapy (tumor significantly shrank after the first course of chemotherapy), or pathological result; and (4) exclusion of coexistence with other sellar/suprasellar lesions or presentation of metastases.

The neuroendocrine disturbance was demonstrated by the pituitary-target hormone profile, which included basal adrenocorticotropic hormone, cortisol, thyroid-stimulating hormone, free triiodothyronine, free thyroxine, gonadotropin, testosterone, estradiol, prolactin, growth hormone, and insulin-like growth factor-1 (IGF-1). Central diabetes insipidus (CDI) was defined as elevated plasma osmolality (≥300 mOsm/kg∙H2O) and hypoosmolar urine (≤200 mOsm/kg.H2O). Short stature was defined as height below -2 standard deviation score by gender and age based on population data of Chinese children. Delayed puberty is defined as the absence of any pubertal signs at the age of 13 years in girls and 14 years in boys. Hypogonadism was defined as amenorrhoea in women and the presence of hypogonadal symptoms along with low serum testosterone levels in men.

## 3. Results

### 3.1. Case Series

**Figure 1:**
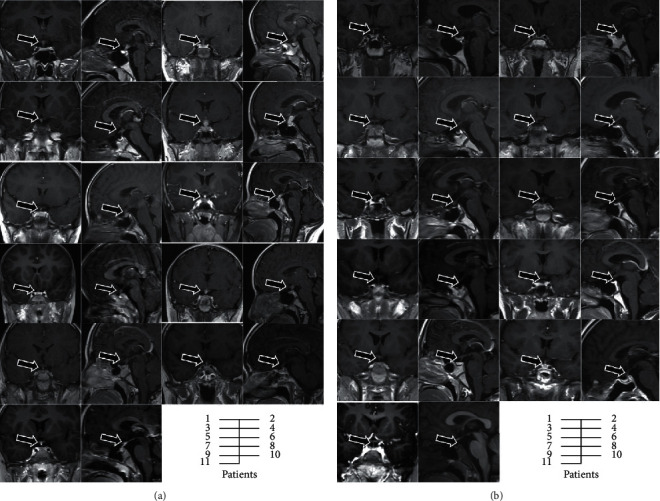
Contrast-enhanced brain magnetic resonance imaging of patients: (a) coronal/sagittal-enhanced brain magnetic resonance imaging at diagnosis and (b) coronal/sagittal-enhanced brain magnetic resonance imaging after chemoradiotherapy (tumor is marked with the arrow).

Seven boys and four girls with an average age of 10.6 ± 4.2 years (range, 5–17) were included in this study. All patients presented with polydipsia and polyuria at the first outpatient visit, with a median symptom interval of 10 (range, 2–48) months (see Table 1). Furthermore, four patients presented with short stature, four with fatigue, one with secondary amenorrhoea, and one with delayed puberty. At the initial MRI scan, marked enhancement of the pituitary stalk on postcontrast T1-weighted MRI with an absent posterior pituitary bright spot was observed. No signs of multiple lesions or metastasis were reported. The average maximum diameter of the pituitary stalk was 5.2 ± 1.6 (range, 3.4–8.6) mm in the patients enrolled. Four patients had a maximum diameter at the upper site of the pituitary stalk, while seven had a maximum diameter at the middle site (see Table 1). Slight optic chiasma involvement was present in one patient, but no visual change occurred (patient 1, see Figure 1). All patients received the detection of tumor markers, at least hCG and AFP, in serum, and three of them synchronously received the CSF test. Seven patients showed an increase in serum hCG and/or AFP levels, and three of them also showed synchronously higher CSF levels. Nine patients, including seven with elevated hCG and two with normal hCG levels, were clinically diagnosed with iGCTs through a good response to chemotherapy. Another two patients with negative tumor markers were diagnosed with germinomas by pituitary stalk biopsy.

Further laboratory assessment indicated that nine patients had concomitant partial anterior pituitary hormone deficiency. Six had impairments to the growth hormone/insulin-like growth factor (GH/IGF) axis, six had hyperprolactinemia, and two had hypogonadotropic hypogonadism. No case involving a deficiency of adrenocorticotropic hormone or central hypothyroidism was reported (see Table 2). Except for hypernatremia related to uncontrolled CDI, biochemical and hematological tests, including blood cell count, electrolyte, kidney, and liver function tests, were almost normal. After being diagnosed, the patients received radiation therapy combined with adjuvant chemotherapy in the oncology department, and all achieved complete responses after chemoradiotherapy (see Figure 1). Re-evaluation of the neuroendocrine function indicated that CDI and impairments to the GH/IGF axis persisted, although the tumor had been eliminated (see Table 2). Three patients with hyperprolactinemia at diagnosis spontaneously normalized, and two remained elevated. For two patients with hypogonadotropic hypogonadism before, a girl restored menstruation (patient 10) while another girl remained in a state of delayed puberty (patient 5). A boy who reached age 14 years at the end of chemoradiotherapy was also considered to have delayed puberty secondary to hypogonadotropic hypogonadism (patient 4).

Ten patients received a follow-up visit for a median period of 1 year (range, 1–4). At the last visit, all patients maintained a complete response, but neurohypophysis dysfunction, presenting as polydipsia and polyuria, persisted. Four patients (40%) had concomitant partial hypopituitarism (see Table 2). Only one of the four patients with low concentrations of GH and IGF-1 developed short stature (patient 4), although the other patients also presented with growth retardation. Three patients had delayed puberty at the last visit (patients 4, 5, and 8). Only one patient developed new central hypothyroidism and constant hyperprolactinemia (patient 4).

### 3.2. Literature Review

A literature search was performed in the PubMed/Medline electronic databases for articles published before September 1, 2021, applying the following search terms: pituitary stalk thickening, germinomas, and intracranial germ cell tumors. The criteria for papers to be included in this study were as follows: (1) patients were definitively diagnosed with iGCT by pathology or good response to chemoradiotherapy; (2) brain imaging revealed isolated pituitary involvement, with a description of pituitary function; (3) studies published in English; and (4) exclusion of reviews, expert opinions, or clinical guidelines. In addition, clinical trials with the purpose of analyzing the etiology of pituitary stalk lesions without neuroimaging details were also excluded.

A total of 16 original articles involving 28 patients were included [9–24] (Table 3), with an average age of 13.4 ± 7.6 (range, 4.5–40) years. Fifteen of them were females, five were males, and the remaining eight patients were of unclear gender. Polydipsia and polyuria were the most common initial manifestations of cases included, followed by short stature, hypogonadism, including delayed puberty and amenorrhoea, and other atypical manifestations related to hypopituitarism such as fatigue and loss of appetite. In addition to CDI, hypopituitarism was observed in 68% of the patients, including growth hormone and IGF-1 deficiency, hypogonadotropic hypogonadism, central hypothyroidism, hyperprolactinemia, and cortisol deficiency. The primary neuroimaging features are a thickening pituitary stalk with an absent bright spot in the posterior pituitary gland, followed by the combination of an enlarged pituitary or sellar/suprasellar change. Only three patients had elevated hCG levels in the CSF, and the other had elevated serum hCG levels. No cases of elevated AFP levels were reported. Among all patients included, only four were directly diagnosed with germ cell tumors through biopsy and pathology results. Nine patients chose to wait, and their extended lesion on rescan MRI or an increase in the hCG level indicated possible germ cell tumors after a period of observation. They were further diagnosed as iGCTs on pathology. In addition to patients who received observation, the other 15 patients were initially misdiagnosed as having other pituitary stalk lesions by CSF examination or biopsy testing; ten had lymphocytic hypophysitis, three had granulomas, and one had Langerhans cell histiocytosis. Among those patients, nine received a high dose of glucocorticoids as an anti-inflammatory treatment for pathologically autoimmune diseases, but their lesions showed no significant regression on MRI. Four patients received growth hormone replacement for short stature, but it also promoted tumor progression. Rescan magnetic resonance imaging indicated an enlarged lesion in these patients and even intracranial metastases. Reassessment of tumor markers was performed in twelve patients, eight of whom presented with increased hCG levels, and the remaining four patients were still negative as before. Finally, all patients, except one, were pathologically diagnosed with iGCTs. The other patient with an elevated CSF hCG level was diagnosed with germinomas through a good response to chemoradiotherapy. Twenty patients reported sequelae of neuroendocrine disturbance after chemoradiotherapy; eight patients reported hormone replacement or pituitary hormone deficiency without details; and the remaining 12 patients primarily presented with persistent CDI, followed by central hypothyroidism, adrenocorticotropic and basal cortisol deficiency, growth hormone and IGF-1 deficiency, and hypogonadotropic hypogonadism.

## 4. Discussion

IGCTs with isolated pituitary stalk involvement are rare. IGCTs have a typical midline distribution, and the sellar region was regarded as the second primary site of iGCTs occurrence, following the pineal region. Even among sellar iGCTs, isolated pituitary stalk lesions are rare [13, 16, 17, 19, 21, 24, 25]. Bifocal lesions with a pituitary stalk and the pineal region or basal ganglia region are more easily valued, while isolated pituitary stalk lesions may be ignored in the early stage. As a malignancy, delayed diagnosis can increase the risk of progression and even metastasis [10, 11]. Given that the initial clinical manifestations usually involve neuroendocrine disorders, multidisciplinary teams are highlighted to pay more attention to the neuroendocrine management of iGCTs with isolated pituitary stalks. To our knowledge, the present study involving 11 patients is the largest series of iGCT patients with isolated pituitary stalk involvement.

The patients included in the present case series and the review studies were mostly children and young adolescents, with no significant differences in sex. Unlike other suprasellar masses with a significant mass effect, presentations related to severe headache, persistent vomiting, visual impairment, disorders of movement, and cognitive decline have rarely been reported. Polydipsia and polyuria are the most common clinical manifestations that occur in almost all patients. Other common manifestations included growth retardation, hypogonadism, atypical fatigue, and loss of appetite. The longest symptom interval is related to polydipsia and polyuria coexisting with growth retardation. Neuroimaging is highly recommended for further etiological diagnosis. The most important neuroimaging predictor of germinomas is an absent signal of the posterior pituitary with a well-enhanced lesion on postcontrast T1-weighted MRI, perhaps presenting with a homogeneous solid mass, irregular cystic mass [17, 19], and thickening pituitary stalk. However, regarding the physiological variation in the size of the pituitary stalk, it seems difficult to diagnose these faintly visible lesions of the pituitary stalk. Most studies evaluating pituitary stalk imaging consider a maximum diameter equal to or greater than 2.5-3 mm as thickening [12, 26, 27], 4 mm at the optic chiasm, 3 mm at the pituitary insertion, or both as pathological thickening [8]. In the present study, the mean diameter of the pituitary stalk was 5.2 ± 1.6 mm as neoplastic diseases, which is commonly larger than nonneoplastic lesion [5, 6, 28]. Although elevated hCG and AFP levels in serum and CSF are the specificity of iGCTs, the problem of low sensitivity remains in the early stage of iGCTs. In this study, only seven patients had significantly elevated hCG levels, and most of the cases with delayed diagnoses in published studies also had initially negative tumor markers. From present cases and published studies, a delayed diagnosis of iGCT with isolated pituitary stalk involvement may be related to (1) a lack of awareness of neuroimaging features of pituitary stalk thickening caused by neoplastic diseases, (2) overestimation of the sensitivity of tumor markers, and (3) the initial pathological results of lymphocytic hypophysis or granulomatous disease leading to misdiagnosis [20, 21, 23]. Early diagnosis and appropriate treatment of iGCT before the occurrence of giant mass effect and metastasis is a key factor for reducing mortality and complications. For patients with manifestations but atypical neuroimaging and/or negative tumor marker, CNS MRI and tumor marker tests should be re-evaluated within a shorter interval than regular observation. For patients with typical neuroimaging, assessing the response of tumors to chemoradiotherapy is highly recommended due to its great effect and relative safety. Although some studies suggested that patients with a pituitary stalk of 6–7 mm receive a pathological examination [8], whether to perform a surgical biopsy deserves careful weighing. On the one hand, although safe [29], pituitary biopsy is a difficult operation, especially for a small lesion, clinical diagnosis based on significant tumor size regression after a low dose of irradiation or a short course of chemotherapy is easier to perform. [30, 31] On the other hand, partial cases of iGCTs were pathologically diagnosed as hypophysitis [10, 11, 13, 17–20, 24] or granuloma disease [9, 15] at the initial biopsy due to the immune response of the host to germinomas. Hence, for patients diagnosed with autoimmune diseases who do not respond well to glucocorticoids, iGCTs should be taken into consideration.

There is not much controversy about the therapeutic strategy in local iGCTs, using a reduced dose of whole-brain or whole-ventricle radiation with adjuvant chemotherapy. Tumors shrank on MRI, and tumor markers normalized indicated remission. Evaluation of the neuroendocrine function after chemoradiotherapy indicated that most of the pituitary defects of the survivors had occurred before therapy rather than newly developed. Meanwhile, the neuroendocrine function of survivors with isolated pituitary involvement is better than that of most sellar/suprasellar lesions. In the present study, only one of the six patients with impairments to the GH/IGF axis, developed short stature at the last outpatient visit. This patient refused to receive a growth hormone supplement due to the uncertain risk of recurrence. For patients with gonadotropin deficiency before, menstruation or erection may sometimes be spontaneously relieved in adults and adolescents. Hyperprolactinemia usually normalizes spontaneously after tumor elimination. Only one patient presented with panhypopituitarism following biopsy, craniospinal plus primary boosting radiotherapy combined with six chemotherapy courses. In this case, the cause of panhypopituitarism may come from the lesion itself or treatment-related damage. Combined with a systematic review of previously published studies, the grade of anterior pituitary hormone deficiency varied greatly, and the risk factors are still unclear, while CDI usually persists. Overall, the neuroendocrine disturbance caused by isolated pituitary stalk iGCTs, as well as manifestations caused by a hypothalamus-pituitary hormone deficiency, are still milder than those caused by other sellar iGCTs. Therefore, whether isolated pituitary stalk involvement could be used as an independent risk factor for predicting long-term neuroendocrine function in patients with iGCT is worth further exploration. With the increasing awareness of this rare disease, future studies may try to take pituitary stalk involvement as a separate subtype of image classification to optimize the prediction of the neuroendocrine outcomes of patients with sellar/suprasellar iGCTs on the classification of initial neuroimaging.

In conclusion, children with diabetes insipidus should be advised to receive enhanced MRI, whether they coexist with hypopituitarism. A hyperintense pituitary stalk with an absent bright signal of the posterior pituitary is a strong signal of neoplasms. Elevated levels of hCG and/or AFP in serum or cerebrospinal fluid indicated iGCTs; however, the negative results could not exclude the diagnosis. If rescan neuroimaging is also suspected, a referral to the oncology department and an evaluation of the response to chemotherapy or radiation therapy are highly suggested. Surgery biopsy should be weighed carefully to protect neuroendocrine function. Germinomas should also be considered in patients diagnosed with autoimmune diseases without a good response to glucocorticoids. After the tumor is completely removed, partial anterior pituitary function damage can spontaneously reverse, while CDI commonly persists. Current studies suggested that, from the perspective of initial imaging estimation, the neuroendocrine outcome of isolated pituitary stalk involvement may be one of the best among all sellar iGCTs; however, whether isolated pituitary stalk involvement could become an independent imaging classification still needs more research in the future.

## Figures and Tables

**Table 1:** Characteristics of the patients.

No.	Patient	Manifestations (month)	D_max_ (mm)	Sagittal MRI (mm)			Coronal MRI (mm)			Tumor markers		Diagnosis
				OC	M	PI	OC	M	PI	hCG^a/b^ mIU/ml	AFP^a/b^ ng/ml	
1	16M	PD/PU and fatigue (6)	6.5	5.3	5.6	3.7	6.5	5.6	5.2	62.1	66.8	iGCT (tumor marker)
2	6M	PD/PU (18)	4.7	3.0	4.4	3.1	3.4	4.7	2.2	0.1	1.25	iGCT (chemotherapy)
3	8M	PD/PU (2)	4.3	2.9	2.2	1.7	4.3	2.2	0.9	0.1	1.22	iGCT (chemotherapy)
4	13M	PD/PU and SS (48)	4.3	3.7	4.0	2.5	2.2	4.3	2.2	0.1	1.82	Germinoma (pathology)
5	13F	PD/PU, DP, and SS (10)	8.6	7.0	6.6	5.0	6.9	8.6	3.4	42.51	1.71	iGCT (tumor marker)
6	9F	PD/PU (12)	4.7	3.6	4.6	3.6	3.8	4.7	3.8	204	1.63	iGCT (tumor marker)
7	5M	PD/PU and fatigue (6)	6.5	4.9	6.5	4.2	1.8	3.6	3.1	11.52	1.71	iGCT (tumor marker)
8	12F	PD/PU and SS (10)	6.6	3.9	4.2	2.3	4.2	6.6	2.8	26.11/26.61	0.605/1.36	iGCT (tumor marker)
9	12M	PD/PU and fatigue (4)	3.9	1.5	3.0	1.9	2.6	3.9	2.6	6.64/22.65	1.55/2.15	iGCT (tumor marker)
10	17F	PD/PU, fatigue, and amenorrhoea (24)	3.4	3.4	1.8	1.1	3.3	1.9	1.4	2.51	1.39	Germinoma (pathology)
11	5M	PD/PU and SS (9)	3.9	3.9	2.8	2.6	3.6	2.7	2.3	14.8/34.72	2.16/2.13	iGCT (tumor marker)

Patient: *M*, male; F, female; manifestations: PD/PU, polydipsia and polyuria; SS, short stature, DP, delayed puberty; HH, hypogonadotropic hypogonadism; MRI: *D*_max_, maximum diameter of the pituitary; OC, optic chiasma; *M*, median of the pituitary stalk; PI, pituitary insertion; tumor markers: ^a^, tumor markers in serum; ^b^, tumor markers in cerebrospinal fluid; hCG. human chorionic gonadotropin; AFP, alpha-fetoprotein; diagnosis: iGCT, intracranial germ cell tumor (without pathological type).

**Table 2:** Neuroendocrine disturbance of the patients.

No.	Patient	Neuroendocrine disturbance		
		Pre-CRT	Post-CRT	Final follow-up (age/year)
1	16M	CDI, HPA, HPRL, and GH/IGF	CDI, HPRL, and GH/IGF	CDI and GH/IGF (21)
2	6M	CDI	CDI	CDI (11)
3	8M	CDI and HPRL	CDI	CDI (11)
4	13M	CDI, GH/IGF, and HPRL	CDI, GH/IGF, HPG, and HPRL	CDI, GH/IGF, HPG, HPT, and HPRL (15)
5	13F	CDI, GH/IGF, and HPG	CDI, GH/IGF, and HPG	CDI, GH/IGF, and HPG (15)
6	9F	CDI and HPRL	CDI	CDI (11)
7	5M	CDI	CDI	CDI (7)
8	12F	CDI and GH/IGF	CDI and GH/IGF	CDI, GH/IGF, and HPG (14)
9	12M	CDI and HPRL	CDI	CDI (14)
10	17F	CDI, GH/IGF, and HPG	CDI and GH/IGF	CDI (18)
11	5M	CDI, GH/IGF, and HPRL	CDI and GH/IGF	-

Patient: M, male; F, female; neuroendocrine disturbance: CDI, central diabetes insipidus; HPA, hypothalamic pituitary adrenal axis; HPG, hypothalamic pituitary gonadal axis; HPT, hypothalamic pituitary thyroid axis; HPRL, hyperprolactinemia, GH/IGF, growth hormone/insulin-like growth factor axis; CRT, chemoradiotherapy.

**Table 3:** Cases of intracranial germ cell tumors with pituitary stalk involvement in the published literature.

Study	Patient	Manifestation (duration)	Neuroendocrine disturbance	Initial MRI	Initial tumor markers	Initial diagnosis (method)	Initial therapeutic strategy (observation duration)	Aggravated manifestations	Repeat MRI	Repeat markers	Diagnosis (method)	Neuroendocrine disturbance
Mooth 1997 [13]	5.8F	PU/PD (5 months)	CDI, normal	Normal	Normal	NA	(8 months)	NA	PST and pineal mass	Normal	GE (pathology)	NA
	10.5F	PU/PD and SS (4 years)	CDI and GHD	Normal	Normal	NA	(3 months)	NA	PST and sellar mass	Elevated CSF hCG	GE (pathology)	NA
	10.5M	PU/PD and SS (2 months)	CDI, GHD, and HPRL	PST	Normal	NA	(9 months)	NA	PST and suprasellar mass	Elevated CSF hCG	GE (pathology)	NA
	11.8F	PU/PD and SS (2 months)	CDI and HPA	PST	Normal	Granuloma (pathology)	(11 months)	NA	Extension of the lesion	Normal	GE (pathology)	NA
	12.9F	PU/PD (25 months)	CDI, HPG, HPT, HPA, and HPRL	PST	Normal	NA	(14 months)	NA	Progressive PST	Normal	GE (pathology)	NA
	18.1F	PU/PD and DP (17 months)	CDI, HPA, HPG, GHD, and HPRL	PST	Normal	NA	(8 months)	NA	NA	Elevated CSF hCG	GE (pathology)	NA

Bettendorf 1999 [10]	8F	PU/PD, SS, and loss of appetite (2 years)	CDI, HPG, GHD, HPT, and HPRL	PST and optic chiasma involvement	Normal	LYH (pathology)	Dexamethasone(18mg/d) for 5 months (5 months)	VI, headache, and vomiting	Multiple intracranial lesions	NA	GE (pathology)	CDI, HPA, HPT, and GHD
Fehn 1999 [11]	12F	PU/PD and SS (2 years)	CDI, HPG, GHD, HPT, and HPRL	PST and enlarged pituitary	Normal	LYH (pathology)	Dexamethasone for 5 months (1 year)	VI, headache, and vomiting	Multiple intracranial lesions	NA	GE (pathology)	CDI and panhypopituitarism
Leger 1999 [14]	13	PU/PD and SS (3.8 years)	CDI	PST, enlarged pituitary, and sellar mass	NA	-	(1.7 years)	NA	PST and enlarged pituitary	NA	GE (pathology)	GHD, HPA, HPT, and HPG
	7	PU/PD and SS	CDI	PST	NA	NA	(1.1 years)	NA	PST and enlarged pituitary	NA	GE (pathology)	GHD, HPA, and HPT
	13	PU/PD and SS (2 years)	CDI	PST and enlarged pituitary	NA	NA	(6 months)	NA	PST and enlarged pituitary	NA	GE (pathology)	GHD, HPA, HPT, and HPG
	8	PU/PD and SS	CDI	PST and enlarged pituitary	NA	NA	(1.8 years)	NA	PST and enlarged pituitary	NA	GE (pathology)	GHD

Endo 2002 [15]	12M	PU/PD, fatigue, and loss of appetite	CDI and panhypopituitarism	Suprasellar and intrasellar mass with optic chiasma involvement and cavernous sinus invasion	Normal	Granuloma (pathology)	Glucocorticoid for 6 months	PU/PD and progressing VI	Relapse and extension	NA	GE (pathology)	Hormone replacement without details
Prosch 2006 [16]	9.5F	PU/PD, vomiting, and fatigue (1year)	CDI and GHD	PST and absent posterior pituitary	Normal	LCH (clinical)	GHRT for 7 months, prednisone (40mg/m^2^), and vinblastine for 11 months (24 months)	NA	PST and enlarged pituitary	NA	GE (pathology)	CDI and panhypopituitarism
Ozbey 2006 [18]	24F	Polymenorrhea and headache	HPT, HPRL, and HPG	PST and suprasellar and intrasellar mass	Elevated serum hCG	LYH (clinical)	Glucocorticoid for 3 weeks (3 months)	NA	No regression	Elevated serum hCG	GE (pathology)	Panhypopituitarism
Mikami-Terao 2006 [17]	13F	PU/PD, headache, SS, fatigue, and DP (2 years)	CDI, HPG, HPT, and GHD	PST and absent posterior pituitary	Normal	LYH (pathology)	Prednisolone (1mg-0.32/kg/d) for 3 months (12 months)	VI	Enlarged mass in the pituitary stalk and hypothalamus	Normal	GE (pathology)	CDI, HPA, HPT, HPG, and GHD
Edouard 2009 [19]	10F	PU/PD, headache, and DP (1year)	CDI, HPT, and GHD	PST and absent posterior pituitary	Normal	LYH (pathology)	GHRT for 5 months (9 months)	NA	Enlarged mass in the pituitary stalk and hypothalamus	NA	GE (pathology)	CDI and HPT
	4.5M	PU/PD and SS (1 year)	CDI and GHD	Isolated PST	Normal	LYH (pathology)	GHRT for 3 years (6 years)	CDI, GHD, HPA, and HPT	Progressive PST	Normal	GE (pathology)	CDI, HPA, HPT, and GHD
Jevalikar 2012 [21]	10M	PU/PD (6 months)	CDI and GHD	Absent signal of posterior pituitary	Normal	LYH (pathology)	GHRT for 2 months (16 months)	Headache and Parinaud's sign	Enlarged infundibular, pineal mass	Elevated CSF hCG	GE (pathology)	CDI, HPA, HPT, and GHD
Guzzo 2012 [23]	24F	PU/PD, amenorrhoea, and fatigue (1 year)	CDI, HPA, HPT, HPG, and HPRL	PST and suprasellar mass	Normal	LYH (pathology)	NA	NA	NA	NA	GE (pathology)	NA
Terasaka 2012 [20]	40F	PU/PD, amenorrhoea, polygalactia, headache, and VI (3 years)	CDI, HPA, HPT, and HPG	PST and intrasellar mass	Normal	LYH (pathology)	Hydrocortisone (1000mg with gradient decrease) for 2 weeks	Deteriorated	Enlarged mass	NA	GE (pathology)	HormoNAl replacement without details
Robison 2013 [12]	5	PU/PD	CDI	Isolated PST	Normal	LYH (pathology)	NA (6 years)	NA	NA	Elevated CSF hCG	GE (pathology)	APD
	19	PU/PD	CDI and hypopituitarism	Isolated PST	Elevated CSF hCG	—	—	—	—	—	GE (pathology)	NA
	10	PU/PD	CDI	Isolated PST	Normal	—	—	—	—	—	GE (pathology)	APD
	11	PU/PD	CDI	Isolated PST	Normal	—	—	—	—	—	GE (pathology)	APD

Zilbermint 2014 [22]	13F	PU/PD, fatigue, and amenorrhoea (1 year)	CDI, HPA, HPT, HPG, and HPRL	PST and extending to hypothalamus	Normal	—	—	—	—	—	GE (pathology)	CDI, HPA, and HPT
Graaf 2020 [9]	12F	PU/PD (3 years)	CDI, HPA, HPT, and HPG	PST, sellar-suprasellar mass, and absent posterior pituitary	Elevated CSF hCG	Granuloma (pathology)	Glucocorticoid (1mg/kg/d) for 3 months (36 months)	CDI, VI, headache, and vomiting	Sellar mass and enhancement of the ependyma and pineal gland	Elevated CSF hCG	GE (pathology)	CDI, HPA, and HPG
Dias 2020 [24]	27M	PU/PD (1 year)	CDI	PST, absent posterior pituitary, and pineal cyst	Elevated CSF hCG	Neurohypophysitis (clinical)	Methylprednisolone (500mg/week to 250mg/week) for 3 months (1 month)	Anejaculation	Enlarged PST, absent posterior pituitary, and pineal cyst	Elevated CSF hCG	GE (pathology)	CDI, HPA, HPT, HPG, GHD

Patient: M, male; F, female; manifestations: PU/PD, polydipsia and polyuria; SS, short stature, DP, delayed puberty; HH, hypogonadotropic hypogonadism; VI, visual impairment; neuroendocrine disturbance: CDI, central diabetes insipidus; HPA, hypothalamic pituitary adrenal axis; HPT, hypothalamic pituitary thyroid axis; HPG, hypothalamic pituitary gonadal axis; HPRL, hyperprolactinemia, GHD, growth hormone deficiency; APD, anterior pituitary hormone deficiency; MRI: PST, pituitary stalk thickening; tumor markers: hCG, human chorionic gonadotropin; AFP, alpha-fetoprotein; CSF, cerebrospinal fluid; diagnosis: LYH, lymphocytic hypophysitis; LCH, Langerhans cell histocytosis; GE, germinomas; NGGCT, nongeminomatous germ cell tumors; therapy: GHRT, growth hormone replacement treatment; NA, not applicable.

## Data Availability

The datasets that were used or analyzed during the current study are available from the corresponding authors on reasonable request.
